# Turnaround Time Trends in Cervical Cytology Screening in South Africa, 2005–2023

**DOI:** 10.1002/dc.70170

**Published:** 2026-07-03

**Authors:** Sizeka Mashele, Nditsheni Nethavhakone, Matshediso Mohlala, Peace Ayeni, Johannes Molomo, Judith Mwansa‐Kambafwile, Jaimie Z. Shing, Julia Bohlius, Mazvita Muchengeti

**Affiliations:** ^1^ National Cancer Registry, National Institute for Communicable Diseases, National Health Laboratory Services Johannesburg South Africa; ^2^ Swiss Tropical and Public Health Institute Allschwil Switzerland; ^3^ University of Basel Basel Switzerland; ^4^ Global and Planetary Health Working Group, Institute for Medical Epidemiology, Biostatistics and Informatics Martin‐Luther‐University Halle‐Wittenberg Halle Germany; ^5^ National Health Laboratory Services Johannesburg South Africa; ^6^ National Department of Health Pretoria South Africa; ^7^ Division of Epidemiology and Biostatistics, School of Public Health, Faculty of Health Sciences University of the Witwatersrand Johannesburg South Africa; ^8^ School of Public Health, Health Sciences Faculty University of Cape Town Cape Town South Africa; ^9^ Division of Cancer Epidemiology and Genetics National Cancer Institute Rockville Maryland USA; ^10^ South African DSI‐NRF Centre of Excellence in Epidemiological Modelling and Analysis Stellenbosch University Stellenbosch South Africa

**Keywords:** cervical precancer screening, cytology, laboratory services, pap smear, South Africa, turnaround time

## Abstract

**Background:**

Timely cervical cytology results are crucial for effective screening and early management of precancerous lesions. However, many low‐ and middle‐income countries face delays in turnaround time (TAT). This study assessed TAT for cytology screening results in South Africa from 2005 to 2023 and identified factors associated with TAT delays.

**Methods:**

We conducted a cross‐sectional analysis of public health routine program data from the National Health Laboratory Service for women aged ≥ 15 years. We defined TAT as the time from sample collection to the release of results. The recommended TAT benchmark is 14 days; we defined delays as TAT exceeding 14 days. We used descriptive statistics and multivariable logistic regression to assess TAT trends and factors associated with delays.

**Results:**

From 2005 to 2023, 12,246,041 cervical cytology smears were processed. The median age at screening was 38.8 years (IQR: 31.0–41.1), ranging from 15 to 95 years. Median TAT consistently exceeded 14 days, peaking at 25 days between 2014 and 2019. Delays were significantly associated with location, with higher odds in self‐sufficient (aOR 2.33; 95% CI: 2.32–2.34) and laboratory‐dependent regions (aOR 1.61; 95% CI: 1.61–1.62). Samples processed in referral laboratories had higher odds of delay (aOR 4.08; 95% CI: 4.07–4.10). Seasonal variation also affected TAT delays, with higher odds observed in the third quarter (aOR 1.40; 95% CI: 1.39–1.40) as compared to the first quarter.

**Conclusion:**

Throughout South Africa, cervical cytology TAT remains above the recommended benchmarks. Delays are driven by structural and operational factors. Strengthening laboratory capacity, optimizing workflows, and transitioning to organized screening programs are critical for timely results and improved cervical cancer prevention outcomes.

**Abbreviations**
aOR: adjusted odds ratio
CI: confidence interval
HPV: human papillomavirus
IHC: immunohistochemistry
IQR: interquartile range
LMIC: low‐ and middle‐income countries
NHLS: National Health Laboratory Service
Pap: papanocoulous
SA: South Africa
SD: standard deviations
SSA: sub‐Saharan Africa
TAT: turnaround time
WHO: World Health Organization

## 1 Introduction

Cervical cancer remains a major global public health issue, particularly in low‐ and middle‐income countries (LMICs), where the majority of cases and deaths occur [1, 2]. This type of cancer is largely preventable through effective screening and early treatment of precancerous lesions [1, 3]. In sub‐Saharan Africa (SSA), cervical cancer significantly contributes to the overall cancer burden among women, highlighting ongoing challenges in accessing timely and effective prevention services [4, 5]. South Africa, like other countries in SSA, continues to face a high incidence of the disease, with an age‐standardized incidence rate of 24.7 per 100,000 [6, 7]. The Papanicolaou (Pap) smear screening is acknowledged as one of the most effective public health measures against invasive cervical cancer and is widely employed as a primary strategy for detecting precancerous lesions [8]. The success of these screening programs depends not only on their reach but also on the speed with which results are processed and communicated. Delays in turnaround time (TAT) can compromise screening effectiveness by delaying diagnosis, follow‐up, and treatment, thereby increasing the risk that the disease progresses to an invasive stage.

Countries with well‐organized, population‐based precancer screening programs have successfully reduced cervical cancer incidence and mortality by ensuring the prompt processing and delivery of screening results [9, 10]. In high‐income settings, most cytology results are delivered within the recommended timeframes, typically within 1–2 weeks, facilitating timely clinical decision‐making [9, 11]. In contrast, evidence from LMICs indicates that delays in diagnostic services are common due to health system constraints such as limited laboratory capacity and workforce shortages [12, 13, 14]. Studies from SSA and the United States have demonstrated that targeted interventions to strengthen laboratory systems can improve TATs for diagnostic services [9, 14]. Despite the availability of proven interventions, the timely delivery of screening results remains a significant bottleneck in many settings.

There is a scarcity of large‐scale, nationally representative data on cervical cytology TAT in LMICs, especially within routine public‐sector screening programs. Existing research often focuses on single institutions, specific regions, or limited time frames, thereby limiting its generalizability and usefulness for national‐level planning. Since the early 2000s, South Africa has provided free Pap smear–based cervical precancer screening in public healthcare facilities nationwide, with cytology serving as the primary screening method. Under the national screening policy, women aged 30 years and older were eligible for three lifetime Pap smears at 10‐year intervals. The third and final screening required clinical assessment by a healthcare provider to determine eligibility for continued screening. Because the program was implemented opportunistically rather than through an organized system, some women initiated screening later than age 30 years, resulting in their final screening occurring beyond 50 years of age.

This context provides an opportunity to understand the determinants of cytology TAT, which is crucial for identifying inefficiencies and informing system‐level improvements. Moreover, as many countries transition to human papillomavirus (HPV) DNA testing as the primary screening method, as recommended by the World Health Organization (WHO), baseline assessments of cytology performance remain vital, given cytology's ongoing role in triage and follow‐up care.

In this study, we aimed to conduct a national evaluation of cervical cytology turnaround times (TATs) within a large public‐sector screening program over an extended period, as countries transition to using HPV DNA testing as the primary screening method. Specifically, the study analyzes TAT trends, investigates factors contributing to delays, and identifies health system characteristics that affect the timely delivery of screening results. This assessment is crucial for monitoring progress toward equitable and efficient screening systems, ensuring that the implementation of HPV DNA testing addresses existing delays in screening results and does not perpetuate inequities in access to timely screening outcomes.

## 2 Methods

### 2.1 Study Design, Setting and Data Source

This cross‐sectional study examined routinely collected cervical cytology screening data from the National Health Laboratory Service (NHLS), which delivers diagnostic services to the public healthcare sector across an entire nation. South Africa, classified as an upper‐middle‐income country, possesses a substantial public healthcare system that serves approximately 84% of the population [15, 16]. The NHLS network comprises a coordinated system of primary healthcare facilities, diagnostic laboratories, and specialized cytology laboratories that collectively facilitate cervical cancer screening services nationwide. Over time, the NHLS improved and modernized cervical cytology screening technologies, transitioning from conventional cytology methods used since 2005 to liquid‐based cytology (LBC) introduced in 2016. Between 2016 and 2018, both conventional cytology and LBC were used concurrently during the phased replacement of older laboratory technologies with newer systems. The study period spanned 2005–2023, enabling assessment of long‐term trends in TAT within a large‐scale public‐sector screening program.

### 2.2 Study Population

**FIGURE 1:**
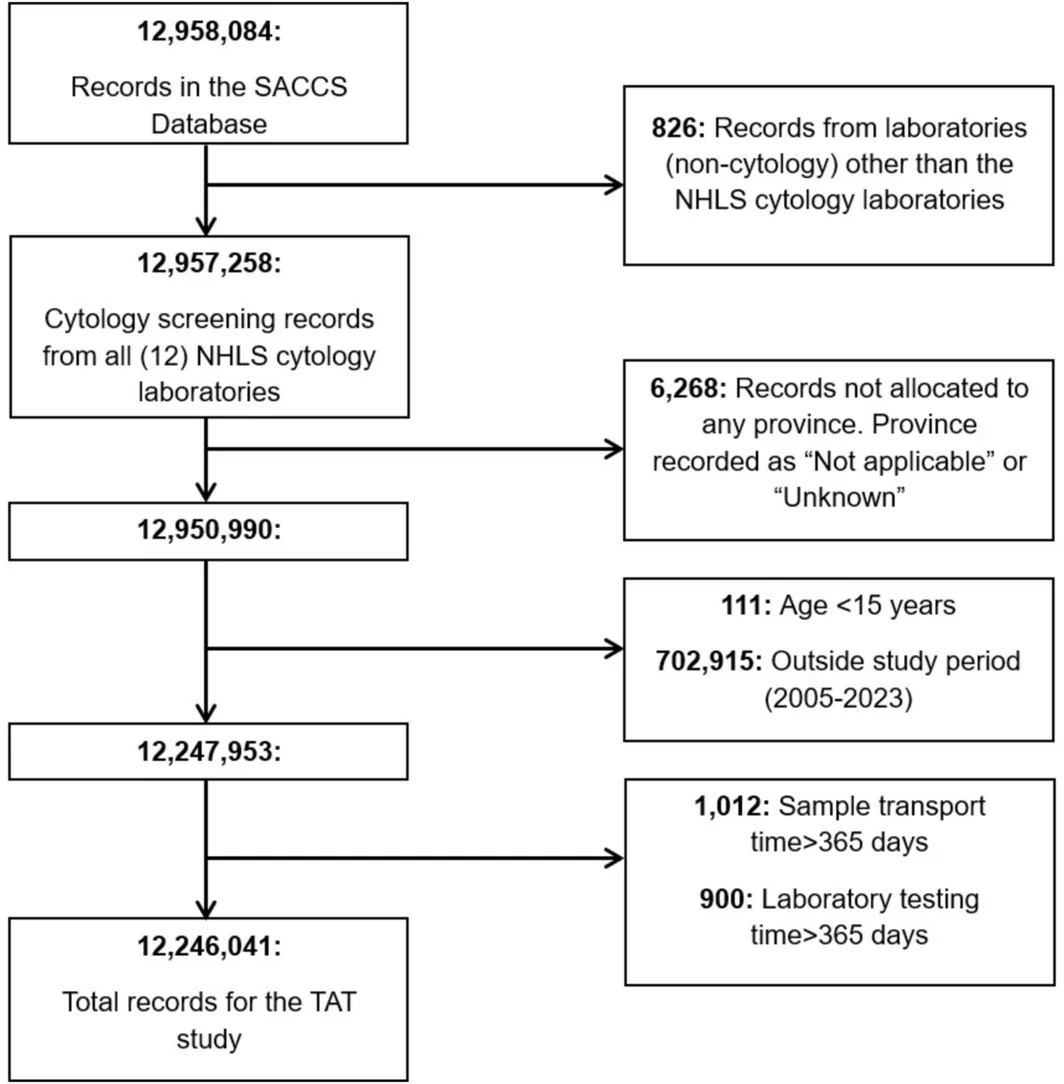
Study participants sampling, 2005–2023.

We included women aged 15 years and older who underwent cervical cancer screening via cytology within the public healthcare system during the specified study period. Records were deemed eligible if a valid cytology screening result was documented in the NHLS database. Records for women under 15 years of age, those screened using non‐cytology methods, and records outside the study period were excluded. The final analytical dataset comprised over 12 million cytology screening records (Figure 1).

### 2.3 Cytology Sample Flow and Laboratory Network Structure

**FIGURE 2:**
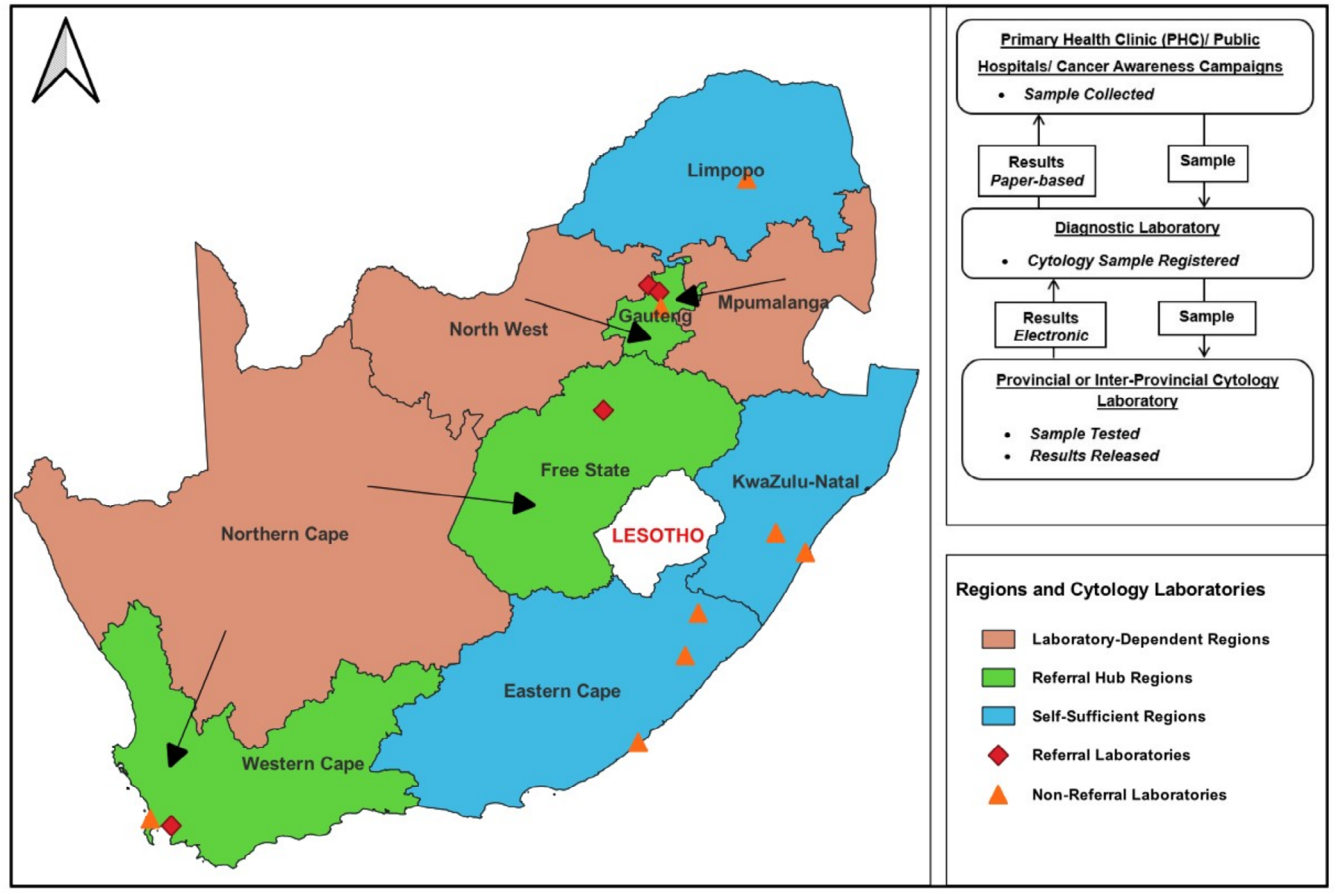
Distribution of public cytology laboratories and cytology sample transfer in South Africa, 2023. [Color figure can be viewed at wileyonlinelibrary.com]

Cervical cytology services are provided through a comprehensive network of public primary health clinics (PHC) and laboratories. Specimens are collected at the primary care level and transported via a centralized laboratory transport system to diagnostic laboratories for registration, followed by processing at designated cytology laboratories. Results are subsequently returned electronically and through established reporting channels to healthcare facilities for patient management (Figure 2).

The laboratory network is structured in tiers, comprising referral laboratories that handle both local and referred specimens and non‐referral laboratories that process exclusively locally collected specimens. Furthermore, we classified regions by their laboratory capacity as (i) laboratory‐dependent regions, which depend on external laboratories for cytology processing; (ii) self‐sufficient regions, which manage locally collected specimens; and (iii) referral hub regions, which process both local and referred specimens (Figure 2).

### 2.4 Data Cleaning and Analysis

Data pertaining to cervical cytology precancer screening were sourced from the NHLS and subsequently verified for completeness and consistency. The variables utilized were those available in the NHLS cytology database, as collected on the cytology request form employed for cervical precancer screening. The data were cleaned and analyzed using Stata version 18.1 SE (StataCorp, College Station, TX, USA). We used descriptive statistics to summarize demographic and laboratory characteristics. We summarized continuous variables using means and standard deviations (SD) or medians and interquartile ranges (IQR), as appropriate. We presented categorical variables as frequencies and percentages.

We categorized the turnaround times (TATs) into three distinct phases: pre‐analytical TAT, which encompasses the duration from sample collection to registration; analytical TAT, which covers the period from sample registration to result release; and total TAT, which spans from sample collection to result release within the NHLS cytology laboratory. We computed the median and interquartile range (IQR) values for TATs for each region and financial quarter. We illustrated trends in TATs across the country using line graphs and evaluated them against international cervical cytology standards.

We defined the binary outcome variable delayed TAT as a total TAT exceeding the international benchmark of 14 days [17]. The exposure variables available for inclusion were age group, HIV status, region, laboratory type, screening context, cytology methods, and seasonal variation. We included all variables in the multivariable logistic regression analysis; however, some were excluded due to collinearity. The variables in the final model were region, laboratory type, screening context, cytology methods, and seasonal variation. We reported adjusted odds ratios (aOR) with 95% confidence intervals (CI). Statistical significance was set at *p* ≤ 0.05.

## 3 Results

### 3.1 Demographic Description of Women Screened for Cervical Cancer

From 2005 to 2023, a total of 12,246,041 cervical cytology screenings were carried out among South African women aged 15 years and older. The median age at the time of screening was 38.8 years, with an interquartile range (IQR) of 31.0–49.1 years, and the ages spanned from 15 to 95 years. Most screenings were conducted on women aged 30–50 years (55.6%), followed by those aged 51 years and above (23.2%), and women aged 20–29 years (19.4%). A smaller percentage of screenings involved adolescents aged 15–19 years (1.8%). HIV status was not recorded for all women; 27.5% reported to be HIV positive and 22.6% reported HIV negative, and the remainder had undisclosed HIV status. In terms of the screening context, 45.6% were part of programmatic services, while 8.7% were due to symptom‐related reasons. Screening activities were evenly distributed throughout the year, with 23.4% in the first quarter, 26.9% in the second quarter, 24.4% in the third quarter, and 25.3% in the fourth quarter (Table 1).

**TABLE 1:** Characteristics of cervical cytology screening records in South Africa, 2005–2023.

Characteristics	Frequency (*n*)	Percentage (%)
Total	*N* = 12,246,041	100
Age group (years)		
15–19	217,402	1.8
20–29	2,375,035	19.4
30–50	6,807,933	55.6
51+	2,845,671	23.2
HIV status		
HIV‐negative	2,770,735	22.6
HIV‐positive	3,368,664	27.5
Undisclosed	6,106,642	49.9
Region		
Referral hub regions	5,576,760	45.5
Laboratory‐dependent regions	1,883,705	15.4
Self‐sufficient regions	4,785,576	39.1
Laboratory type		
Referral laboratories	9,084,158	74.2
Non‐referral laboratories	3,161,883	25.8
Screening context		
Programmatic screening	5,578,954	45.6
Symptom‐driven screening	1,061,173	8.7
Not recorded	5,605,914	45.8
Cytology methods		
Conventional cytology	5,494,711	44.9
Transition period^a^	2,518,781	20.6
Liquid‐based cytology	4,232,549	34.6
Seasonal variation		
First quarter	2,863,394	23.4
Second quarter	3,294,426	26.9
Third quarter	2,993,655	24.4
Fourth quarter	3,094,566	25.3

^a^ The period when laboratories were migrating to liquid‐based cytology (LBC). Regions are classified into (i) laboratory‐dependent regions, which depend on external laboratories for cytology processing; (ii) self‐sufficient regions, which manage locally collected specimens; and (iii) referral hub regions, which process both local and referred specimens.

### 3.2 Cytology Screening Uptake Over Time

**FIGURE 3:**
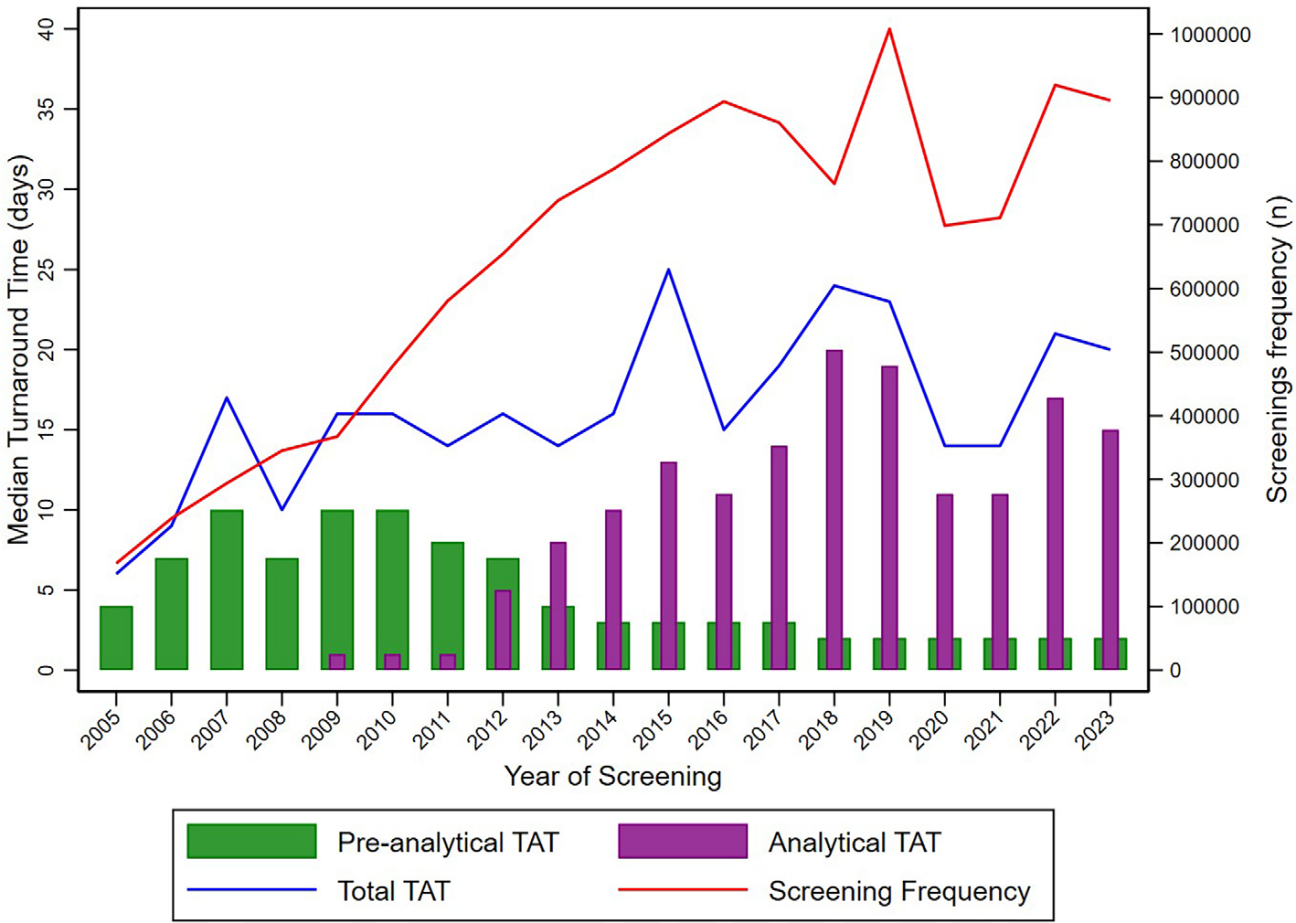
National trend in TATs for cervical cytology screening and the frequency of screens conducted in South Africa, 2005–2023. [Color figure can be viewed at wileyonlinelibrary.com]

Over the course of the study, there was a significant increase in the annual count of cervical cytology screenings. Beginning in the mid‐2000s, the number of screenings rose steadily, reaching a peak of 900,000 by the late 2016s. After this period of growth, the frequency of screenings fluctuated in recent years, with temporary declines followed by a rebound towards the end of the study. Overall, the long‐term trend reflects a persistent rise in screening activities (Figure 3).

### 3.3 Pre‐Analytical TAT for Cytology Screening

During the study period, the pre‐analytical TAT improved significantly. Initially, pre‐analytical TATs were lengthy, with a median duration of 10 days. However, over time, there was a noticeable decrease in pre‐analytical TAT, with sample transport times becoming more consistent and shorter in the later years. This positive change became stable at relatively low levels within 2 days, particularly from early 2013 onwards (Figure 3).

### 3.4 Analytical TAT for Cytology Screening

Throughout the study period, the analytical TAT exhibited significant fluctuations. In the initial years, the analytical TAT was typically brief, with median values nearly at 0 days. However, starting in early 2012, there was a marked increase in analytical TAT, with median values in certain periods reaching approximately 20 days. From approximately 2016 onwards, the analytical TAT began to stabilize gradually, with median values generally falling between 10 and 20 days in more recent years (Figure 3).

### 3.5 Total TAT for Cytology Screening

**FIGURE 4:**
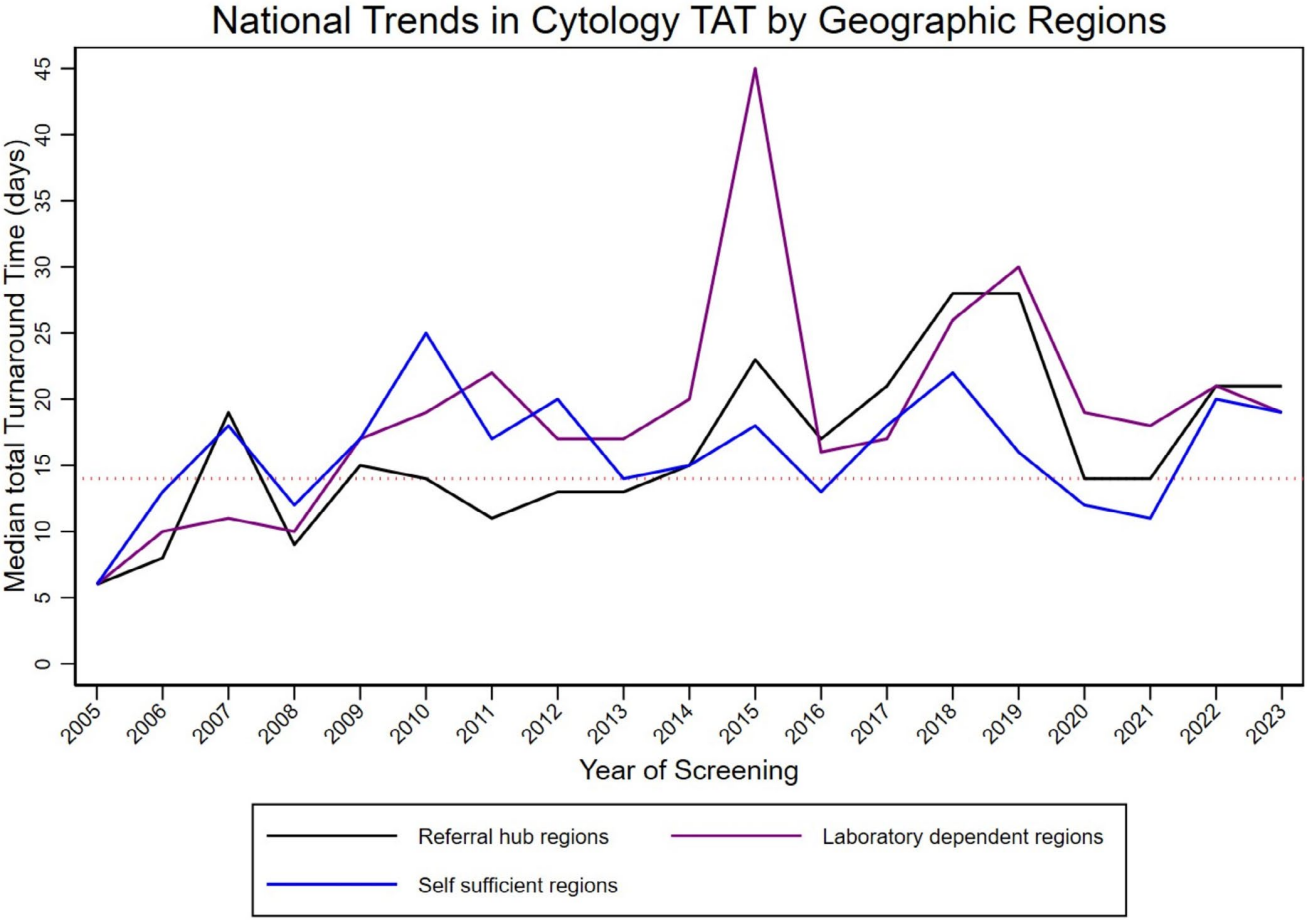
National Trends in total TAT for cervical cytology screenings by geographic region, 2005–2023. [Color figure can be viewed at wileyonlinelibrary.com]

**FIGURE 5:**
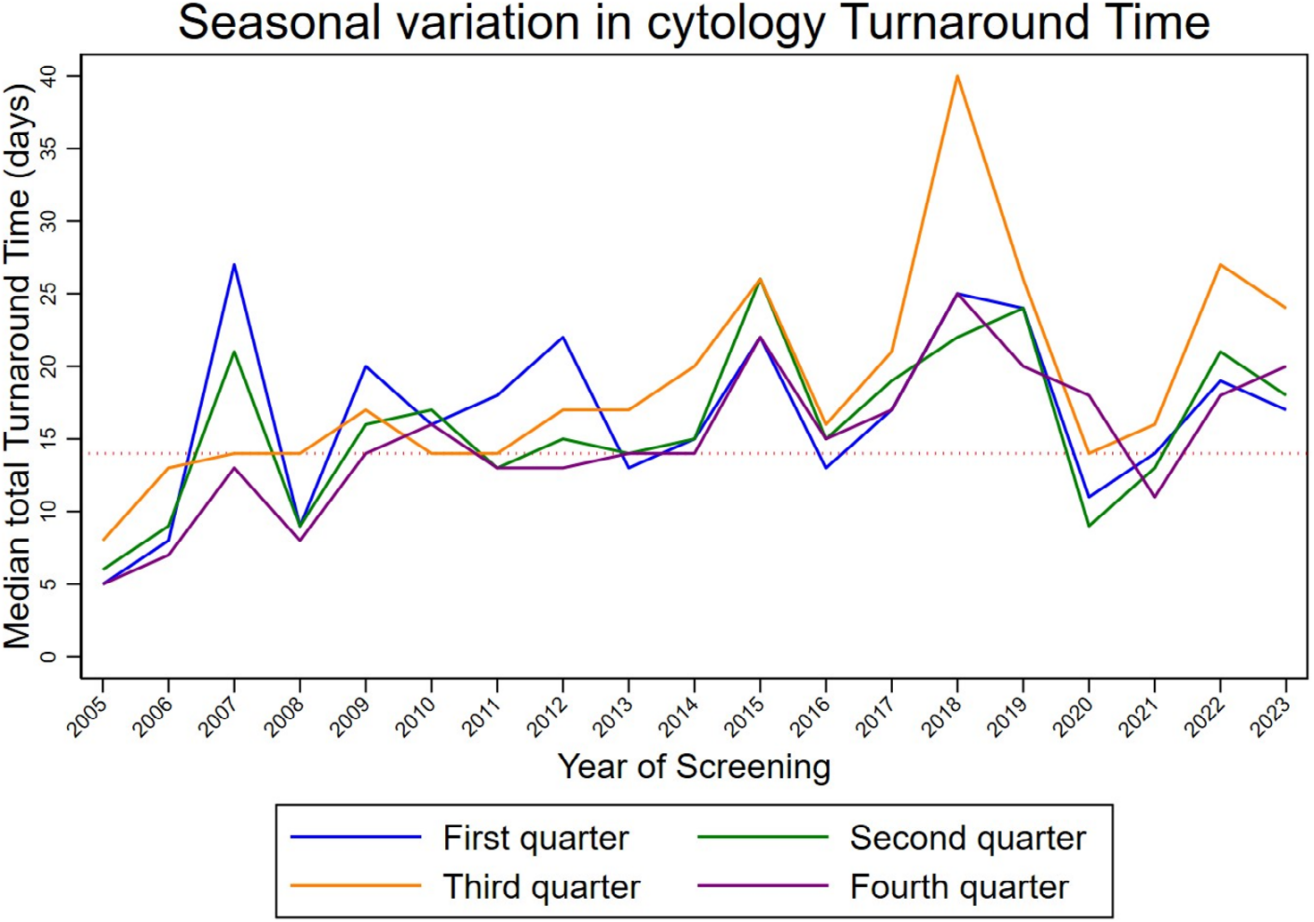
Seasonal variations in cytology TAT in South Africa, 2005–2023. [Color figure can be viewed at wileyonlinelibrary.com]

Throughout the study period, the median total turnaround time (TAT) consistently surpassed the recommended international benchmark of 14 days or less for releasing cytology results. Notably, from 2014 to 2019, there was a marked delay, with the median reaching 25 days (Figure 3). Median total TAT varied across different regions. Laboratory‐dependent regions experienced the longest delays (median = 45 days), with median TATs consistently exceeding the recommended 14‐day benchmark. Similarly, referral hub regions also demonstrated prolonged TATs (median = 25 days). Self‐sufficient regions generally demonstrated shorter median TATs, with values closer to, but still often exceeding, the 14‐day benchmark (Figure 4). Quarterly analysis of TATs revealed regular variations. Starting in 2012, the first quarter consistently had the shortest median TATs, with results generally available in 16 days. The second and fourth quarters experienced slightly longer durations, with a median of 18 days. The third quarter consistently had the longest delays, with the median TAT reaching 40 days (Figure 5).

### 3.6 Factors Associated With Delay in TAT for Cytology Screening Results

Several health system and temporal factors were significantly associated with delayed TAT for cervical cytology screening. Laboratory‐dependent regions (aOR 1.61; 95% CI: 1.61–1.62) and self‐sufficient regions (aOR 2.33; 95% CI: 2.32–2.34) had higher odds of a TAT delay compared to referral hub regions. Samples processed in referral laboratories had over four times higher odds of delayed TAT compared to those processed in non‐referral laboratories (aOR 4.08; 95% CI: 4.07–4.10). Programmatic screening was associated with slightly higher odds of delay compared to symptom‐driven screening (aOR 1.16; 95% CI: 1.16–1.17). Samples processed during the transition to liquid‐based cytology had significantly higher odds of delay (aOR 1.75; 95% CI: 1.75–1.76), as compared to those processed during the liquid‐based cytology period (aOR 1.52; 95% CI: 1.52–1.53). Compared to the first quarter of the year, the odds of delay were higher in the second quarter (aOR 1.06; 95% CI: 1.05–1.06) and peaked in the third quarter (aOR 1.40; 95% CI: 1.39–1.40), while the fourth quarter showed slightly reduced odds (aOR 0.93; 95% CI: 0.93–0.93) of delay (Table 2).

**TABLE 2:** Health system factors associated with delay in cytology screening results in South Africa, 2005─2023.

Characteristics	Frequency *n* (%)	cOR (95% CI)	aOR (95% CI)
Region			
Referral hub regions	5,576,760 (45.5)	1	
Laboratory‐dependent regions	1,883,705 (15.4)	1.63 (1.63─1.64)	**1.61** (1.61─1.62)
Self‐sufficient regions	4,785,576 (49.9)	1.02 (1.01─1.02)	**2.33** (2.32─2.34)
Laboratory type			
Non‐referral laboratories	3,161,883 (25.8)	1	
Referral laboratories	9,084,158 (74.2)	1.99 (1.99─2.00)	**4.08** (4.07─4.10)
Screening context			
Programmatic screening	5,578,954 (45.6)	1.18 (1.18─1.19)	**1.16** (1.16─1.17)
Symptom‐driven screening	1,061,173 (8.7)	1	
Not recorded	5,605,914 (45.8)	0.61 (0.61─0.63)	0.72 (0.71─0.72)
Cytology methods			
Conventional cytology	5,494,711 (44.9)	1	
Transition period^a^	2,518,781 (20.6)	2.04 (2.03─2.04)	**1.75** (1.75─1.76)
Liquid‐based cytology	4,232,549 (34.6)	1.89 (1.88─1.90)	**1.52** (1.52─1.53)
Seasonal variation			
First quarter	2,863,394 (23.4)	1	
Second quarter	3,294,426 (26.9)	1.04 (1.04─1.05)	1.06 (1.05─1.06)
Third quarter	2,993,655 (24.4)	1.33 (1.32─1.33)	**1.40** (1.39─1.40)
Fourth quarter	3,094,566 (25.3)	0.95 (0.94─0.95)	0.93 (0.93─0.93)

*Note:* Bold: statistically significant association at *p* ≤ 0.05.

Abbreviations: aOR, adjusted odds ratio; cOR, crude odds ratio; OR, odds ratio.

^a^ The period when laboratories were migrating to liquid‐based cytology. Regions are classified into (i) laboratory‐dependent regions, which depend on external laboratories for cytology processing; (ii) self‐sufficient regions, which manage locally collected specimens; and (iii) referral hub regions, which process both local and referred specimens.

## 4 Discussion

This study provides a comprehensive national evaluation of cervical cytology TAT utilizing over 12 million screening records from a large public‐sector programme from 2005 to 2023. During the study period, the volume of cytology screenings consistently increased nationwide until 2016, after which a mixed trend was observed with a marked decline in 2020 and 2021, most likely due to the COVID‐19 pandemic. The overall TAT exceeded the recommended 14‐day benchmark in several years, indicating persistent delays in delivering screening results. Cytology TATs are influenced mainly by location and screening volumes, and to some extent by the time of year. Women screened in the regions where there are no specialized cytology laboratories, as well as those screened in the third quarter of the year, experienced the longest delays, with a median of 40 days. Delays in TAT were primarily associated with health system factors, including regions lacking cytology laboratories, centralized sample processing in referral laboratories, and samples collected during the third quarter of the financial year. The third quarter of the financial year has seen an annual increase in pap smear volumes, driven by mass screening campaigns as part of the World Cervical Cancer Elimination Month commemorations.

The findings of this study are consistent with existing evidence demonstrating that delays in diagnostic TAT remain a major challenge in low‐ and middle‐income countries (LMICs). Studies from high‐income settings, such as the Q‐Probes program in the United States, reported that 90% of laboratories release gynaecologic cytology results within 8 days or less, and non‐gynaecologic cytology results within 3 days [11, 18], with most laboratories meeting recommended benchmarks. In contrast, the longer TATs observed in this study reflect systemic constraints commonly reported in resource‐limited settings. Comparable studies from sub‐Saharan Africa (SSA) have also documented prolonged TAT for pathology services. For example, reductions in TAT following targeted interventions have been reported in Botswana [12, 13], while studies from other SSA countries continue to show delays exceeding several weeks [19, 20]. These findings suggest that challenges related to laboratory capacity, workforce limitations, and sample transport systems are widespread across similar health system contexts. Comparable studies also show that screening uptake has increased in Southern Africa over the last two decades, indicating improved access to these screening services and a positive indicator of progress towards reaching the 2030 WHO cervical cancer elimination targets [21].

The results of this study highlight that delays in cytology TAT are largely driven by structural and operational factors within the health system. Centralized laboratory models, while beneficial for quality assurance, may contribute to delays when demand exceeds processing capacity. This underscores the need for optimized laboratory network design, including improved workload distribution and potential decentralization where feasible. The observed seasonal variation and regional variation in TAT further suggest that fluctuations in screening demand can strain laboratory capacity, particularly in settings with opportunistic or campaign‐driven screening approaches. Transitioning toward organized screening programs, with scheduled invitations, predictable demand, and coordinated resource allocation may help stabilize workload and improve TATs. Importantly, the increase in screening uptake over time reflects progress in expanding access to cervical cancer screening services. However, without parallel investments in laboratory infrastructure and efficiency, increased demand may inadvertently contribute to delays in result reporting. As countries transition toward HPV DNA testing, these findings emphasize the importance of strengthening laboratory systems to ensure timely follow‐up and effective program performance.

This study has several notable strengths. To our knowledge, this is the first study in South Africa to evaluate cytology TAT using routine public sector laboratory data, providing essential baseline information for the national cytology screening program. It represents one of the largest evaluations of cytology TAT, providing nationally representative data over an extended period. The use of routinely collected laboratory data enables assessment of real‐world program performance, thereby enhancing the relevance of the findings for health system planning and policy. However, several limitations have been considered. First, the analysis was limited to cytology‐based screening within the public sector and may not be generalizable to private healthcare settings. Second, the study did not capture the time from the release of the laboratory result to patient notification, an important component of the overall care pathway. Third, incomplete data for certain variables, such as HIV status and screening context, may have influenced some estimates.

In conclusion, this study demonstrates that cervical cytology TATs remain above recommended benchmarks in a large public‐sector screening program. Delays are primarily driven by health system factors, including laboratory network structure, workload pressures, and operational inefficiencies. Addressing these challenges will require a systems‐level approach focused on strengthening laboratory capacity, improving workflow efficiency, and transitioning toward organized screening programs. These findings provide important insights for countries seeking to optimize cervical cancer screening systems and ensure timely delivery of results as part of global efforts toward cervical cancer elimination.

## Author Contributions

Sizeka Mashele conceived, conducted, analyzed, interpreted, and wrote the manuscript. Mazvita Muchengeti and Julia Bohlius supervised, advised, and reviewed the manuscript. Nditsheni Nethavhakone provided expertise and reviewed the manuscript. Judith Mwansa‐Kambafwile, Johannes Molomo, Peace Ayeni, Matshediso Mohlala, and Jaimie Z. Shing reviewed results and provided feedback. All authors approved the final draft.

## Funding

This research was supported in part by the Intramural Research Program of the National Institutes of Health (NIH) and the Swiss Cancer Research Foundation (KFS‐5415‐08‐2021). Additional support for SM's PhD stipend was co‐funded by the German Federal Ministry of Research, Technology, and Space, 01KA2220B, and in part by the Science for Africa Foundation to the Developing Excellence in Leadership, Training and Science in Africa (DELTAS Africa) program (Del‐22‐008) with support from Wellcome Trust and the UK Foreign, Commonwealth & Development Office and is part of the EDCPT2 program supported by the European Union and the Vital Strategies Bloomberg Philanthropies Data for Health Initiative. The contributions of the NIH author(s) are considered Works of the United States Government. The findings and conclusions presented in this paper are those of the author(s) and do not necessarily reflect the views of the funders. The funders had no role in the design of the study, data collection, analysis, or manuscript preparation.

## Ethics Statement

This study received approval from the Human Research Ethics Committee at the University of Witwatersrand (Ref. No. M231085 M240422‐A‐0001), NHLS Academic Affairs (reference number PR2346413), and National Cancer Institute Ethics Review Panel (Ref. No. 24G007‐03).

## Consent

All authors approved the final draft for publication.

## Conflicts of Interest

The authors declare no conflicts of interest.

## Data Availability

The data that support the findings of this study are available on request from the corresponding author. The data are not publicly available due to privacy or ethical restrictions.
